# RSV Infection in Human Macrophages Promotes CXCL10/IP-10 Expression during Bacterial Co-Infection

**DOI:** 10.3390/ijms18122654

**Published:** 2017-12-07

**Authors:** Daniela Machado, Jonathan Hoffmann, Marie Moroso, Manuel Rosa-Calatrava, Hubert Endtz, Olivier Terrier, Glaucia Paranhos-Baccalà

**Affiliations:** 1 Laboratoire des Pathogènes Emergents, Fondation Mérieux, Centre International de Recherche en Infectiologie (CIRI), INSERM U1111, CNRS UMR5308, ENS Lyon, Université Claude Bernard Lyon 1, Université de Lyon, 69007 Lyon, France; daniela.bandeira@fondation-merieux.org (D.M.); jonathan.hoffmann@fondation-merieux.org (J.H.); marie.moroso@fondation-merieux.org (M.M.); hubert.endtz@fondation-merieux.org (H.E.); glaucia.baccala@biomerieux.com (G.P.-B.); 2 Virologie et Pathologie Humaine—VirPath Team, Centre International de Recherche en Infectiologie (CIRI), INSERM U1111, CNRS UMR5308, ENS Lyon, Université Claude Bernard Lyon 1, Université de Lyon, 69008 Lyon, France; manuel.rosa-calatrava@univ-lyon1.fr

**Keywords:** respiratory syncytial virus (RSV), *Streptococcus pneumoniae* (*Sp*), macrophages, co-infection, acute lower respiratory infection

## Abstract

Respiratory syncytial virus (RSV), a major etiologic agent of acute lower respiratory infection constitutes the most important cause of death in young children worldwide. Viral/bacterial mixed infections are related to severity of respiratory inflammatory diseases, but the underlying mechanisms remain poorly understood. We have previously investigated the intracellular mechanisms that mediate the immune response in the context of influenza virus/*Streptococcus pneumoniae* (*Sp*) co-infection using a model of human monocyte-derived macrophages (MDMs). Here, we set up and characterized a similar model of MDMs to investigate different scenarios of RSV infection and co-infection with *Sp*. Our results suggest that *Sp* contributes to a faster and possibly higher level of CXCL10/IP-10 expression induced by RSV infection in human MDMs.

## 1. Introduction

The common lower respiratory infections (LRIs), such as pneumonia and bronchiolitis, constitute the major cause of pediatric mortality worldwide but also contribute to high mortality rates among elderly [1] and immunocompromised patients [2,3]. A wide variety of pathogens contribute to LRIs mainly including viruses and bacteria. Studies have underlined the relevance of mixed viral/bacterial infections as etiological risk factors in acute LRIs [4,5] and their impact in severe LRIs. Viral and bacterial co-infections were extensively studied in the context of influenza viral infection, followed by pneumococcal infection [6,7,8,9,10]. However, the mechanisms underlying pathogenesis in mixed infections remain poorly understood, notably in the context of other respiratory pathogens.

Respiratory syncytial virus (RSV) is the most important cause of death in very young children, and global RSV disease burden is estimated in approximately 200,000 deaths and over 3 million hospitalizations per year. Currently, the most common causative agent of viral LRIs is RSV [11,12]. In contrast with influenza viruses, prophylactic and therapeutics approaches for RSV remain very limited and new vaccine and antiviral strategies are urgently required. Among bacterial pathogens, *Streptococcus pneumoniae* (*Sp*) is the most common cause of severe pneumonia. It is a commensal of the nasopharynx and most carriage episodes do not result in disease [13] but pneumococcal pneumonia results in approximately half a million deaths in children younger than 5 years each year worldwide.

A large retrospective study performed recently provided evidence for an interaction between RSV and pneumococcal pneumonia [14]. In children with acute LRIs, RSV was associated with increased colonization by *Sp*, in a similar way to that described in the context of influenza infection [15,16,17,18,19], and although we do not know the exact mechanisms of this interaction, it is assumed that it is due to the increase of attachment sites to the bacteria in the epithelial cells caused by viruses. The prevalence of RSV/*Sp* co-infection varies in approximately 40% of samples from pediatric RSV infections [20,21,22], and in vitro and in vivo studies show significant increases in disease severity in this context [15,23,24,25,26].

Although RSV infections involve several types of cells in the lung, the important role of macrophages in the host response has been demonstrated [27,28,29]. In the lungs, macrophages are strategically located and play a central role in the defense of the host against respiratory pathogens. They initiate innate and adaptive immune responses, besides being the primary source of cytokines and chemokines [30,31]. The elimination of macrophages during a viral or bacterial infection is usually favorable to the pathogen and increases the severity of the disease, suggesting that macrophages are necessary for viral elimination and control of the disease [28,31,32,33]. However, the underlying mechanisms of infection control by macrophages are not fully elucidated in the context of RSV/*Sp* mixed infections.

Recently, we have investigated the intracellular mechanisms that mediate the immune response in the context of influenza virus/*Sp* infection, using a model of human monocyte-derived macrophages (MDMs) [6]. This study revealed that mixed infections of MDMs induces a synergistic production of interferon-γ-induced protein 10 (IP-10/CXCL10). This cytokine could constitute an interesting marker, as it was related to viral and/or bacterial etiologies and disease severity in a retrospective analysis of clinical samples [6]. McNamara and colleagues reported that IP-10/CXCL10 is one the most abundant cytokines in bronchoalveolar lavages (BAL) form infants with RSV bronchiolitis [34]. In the present study, we wanted to set up and characterize a similar model of MDMs to investigate different scenarios of RSV infection and co-infection, focusing on IP10 as a reference indicator of the immune response.

## 2. Results and Discussion

**Figure 1:**
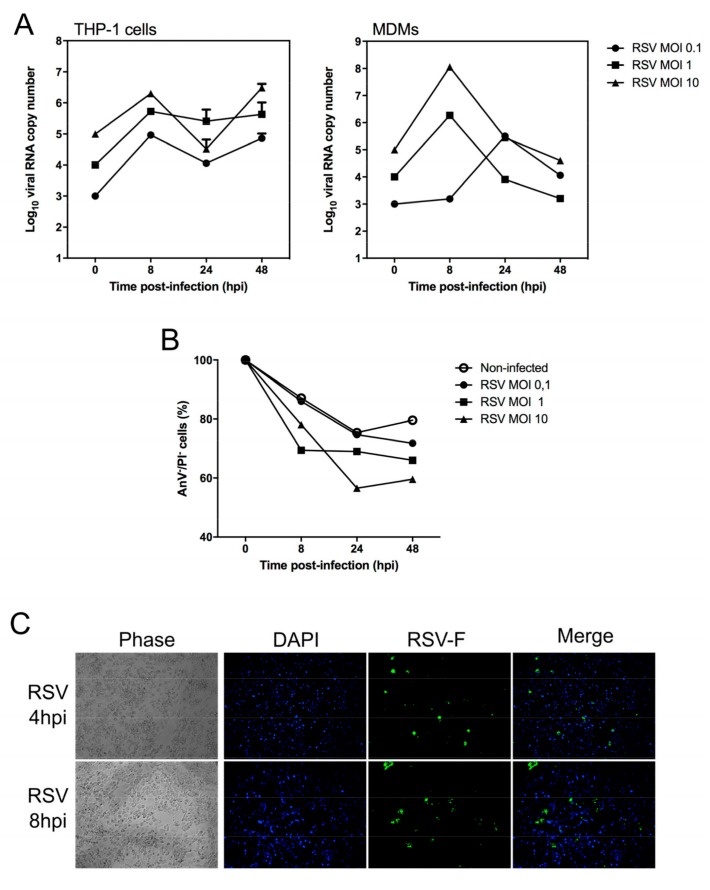
Characterization of RSV infection in human monocyte-derived macrophages (MDMs). Monocytes were isolated from human peripheral blood mononuclear cells (PBMCs) and differentiated into macrophages as previously described (1). RSV (Lon strain, ATCC VR-26) was propagated in HEp2 cells and isolated as described previously [40]. (**A**) THP-1 or MDMs cells were exposed to RSV Long at different multiplicity of infection (MOI, 0.1, 1 and 10) to investigate the kinetics of infection over time. Uninfected control replicates were exposed to cell culture medium. At different time points, total cell extracts were harvested and analyzed by multiplex real-time RT-qPCR to quantify RSV (F fusion gene). The RT-PCR reaction was performed using the AgPath-ID™ One-Step RT-PCR kit (Life Technologies; Carlsbad, CA, USA) according to the manufacturer’s instructions. Samples with a cycle threshold (C*_t_*) value of ≥40 were recorded as negative. A standard curve was prepared using serially diluted RNA extracts from a known quantity and used to calculate genomic copies/mL; (**B**) In the same experimental condition, cell survival in infected MDMs was monitored using flow cytometry, using the FITC/Annexin V apoptosis detection kit (BD Biosciences), according to the manufacturer’s instructions. Percent survival is expressed compared to viable cells measured at *T* = 0 hpi; (**C**) Immunofluorescence microscopy of MDMs infected by RSV (MOI of 1) at 8 hpi. Magnification ×20. Immunofluorescence protocol was performed as previously published [41]. RSV F antigen (Green), Nuclei stained by DAPI (Blue). Each experiment was performed in duplicate, from two separate experiments.

First, monocytes were isolated from human peripheral blood mononuclear cells (PBMC) and differentiated into macrophages as previously described [6]. These human MDMs were then exposed to RSV (long strain, ATCC VR-26) at different multiplicity of infection (MOI) to investigate the kinetics of infection by monitoring of genome copy number using a specific RT-qPCR on cell lysates harvested at different time points (Figure 1A). As a comparison, immortalized monocyte-like THP-1 cells were infected in parallel with the same protocol. Our results confirmed that MDMs can be effectively infected by RSV, similarly to THPI-1, but that viral replication remains very limited in the case of MDMs. These observations were in good agreement with early works that showed that RSV replication in macrophages was possible but to a limited extent [35,36]. Indeed, we observed a similar kinetic profile whatever the MOI used, with a replication peak at 8 hpi, followed by a stabilization or a decrease of genome copy number at 48 hpi for THP-1 and MDMs, respectively (Figure 1A). In contrast to what is usually described in cell lines (e.g., Hep-2 or A549), we did not observe marked cytopathic effects in infected MDMs, but rather a slightly higher number of dying cells. To complete these observations, we then monitored cell death during RSV infection using flow cytometry (Annexin V/propidium iodide staining) (Figure 1B). Our results confirmed that RSV infection increased cell death in MDMs, with a significant decrease of survival at an MOI of 10 at 48 hpi (Figure 1B). In parallel, we performed immunofluorescence microscopy of RSV-infected MDM, using a specific antibody to detect RSV F protein (ab20745; Abcam, Cambridge, UK) (Figure 1C). At an MOI of 1 at 8 hpi, around 10–15% of cell monolayer was positive for RSV antigen staining (Figure 1C), indicating an efficient infection, and possibly an initial limited viral replication, as RSV F staining still progressively increases from early stages to 8 hpi. Interestingly, the level of apoptosis in RSV-infected cells (Figure 1B) is not completely correlated with the number of infected cells (Figure 1C), suggesting that apoptosis is also induced in non-infected cells. Altogether, these results indicate that human MDMs are susceptible to RSV infection. Moreover, viral replication occurs, to a limited extent, in slight disagreement with previous observations indicating abortive infection in lung macrophages [37,38,39].

**Figure 2:**
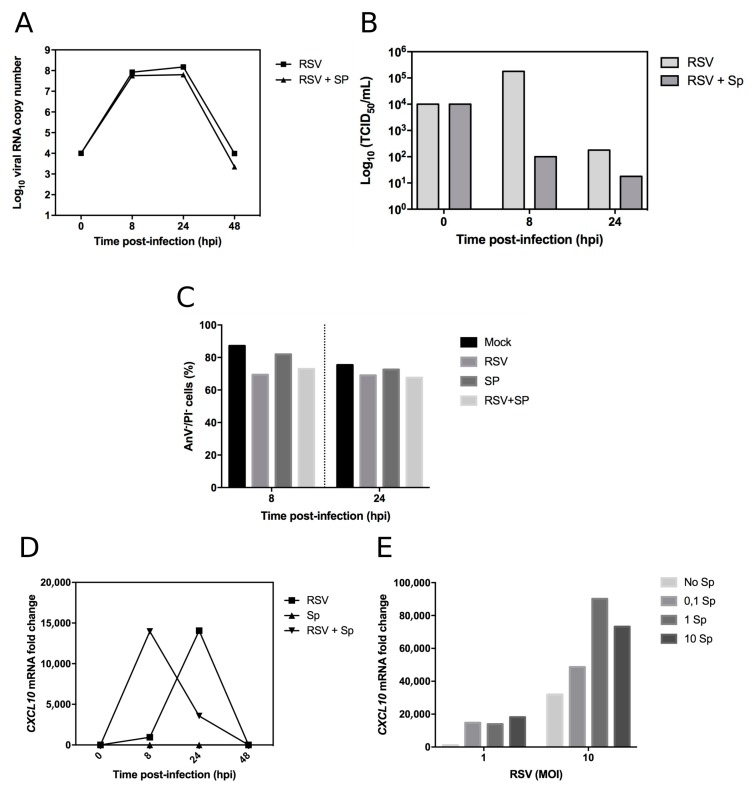
Characterization of RSV/*Sp* mixed infection in human monocyte-derived macrophages (MDMs). Human MDMs were infected by RSV at an MOI of 1. After 4 h of viral infection, cells were mock-infected or infected by *Sp* at 1 CFU/cell. Encapsulated *Streptococcus pneumoniae* serotype 14 was obtained from the National Reference Center for *Streptococci* (Department of Medical Microbiology, Aachen, Germany). *Pneumococci* were opsonized in anti-pneumococcal immune serum and incubated for 30 min at 37 °C prior to infections for all experiments. MDM cells showed significant loss of viability following overnight incubation with *S. pneumoniae*, and gentamicin (10 µg/mL) was therefore added to all wells at the 2-h time point to prevent bacterial overgrowth and loss of cell viability. (**A**) At different time points, total cell extracts were harvested and analyzed by multiplex real-time RT-qPCR to quantify RSV; (**B**) RSV viral production in supernatants was performed by quantification of viral titers (TCID 50/mL) with a limit-dilution assay and using the Reed & Muench statistical method. Results are expressed as log10 TCID50/mL values; (**C**) In the same experimental condition, cell survival in infected MDMs was monitored using flow cytometry, with the FITC/Annexin V apoptosis detection kit (BD Biosciences), according to the manufacturer’s instructions. Percent survival is expressed compared to viable cells measured at *T* = 0 hpi; (**D**,**E**) Co-infection alters the timing and extent of IP-10 expression in human monocyte-derived macrophages (MDMs). Human MDMs were infected by RSV at an MOI of 1. After 4 h of viral infection, cells were mock-infected or infected by *Sp* at 1 CFU/cell. At different time points, total cell extracts were harvested and analyzed by multiplex real-time RT-qPCR to quantify CXCL10 mRNA levels. Total mRNA was purified from transfected and infected MDMs using the RNeasy kit (Qiagen) and specific primers were used to amplify CXCL10 (F: 5′-GTGGCATTCAAGGAGTACCTC-3′, R: 5′-TGATGGCCTTCGATTCTGGATT-3′) and β-Actin for normalization (F: 5′-CTCTTCCAGCCTTCCTTCCT-3′, R: 5′-AGCACTGTGTTGGCGTACAG-3′); (**E**) Similar strategy with different combinations of RSV and *Sp*, at 8 hpi. Each experiment was performed in duplicate, from two separate experiments.

In a second step, we used this MDM model to study RSV/*Sp* mixed infection. We have previously demonstrated that human MDMs were permissive to *Sp* infection [6]. Using a similar experimental approach, MDMs were first infected by RSV at an MOI of 1 and then mock-infected or infected by *Sp* at 1 CFU/cell, 4 h after viral infection. Viral kinetics were monitored by RT-qPCR for 48 hpi (Figure 2A) and by measure of RSV viral load (TCID50/mL) in the supernatants (Figure 2B). Whereas the genome copy number remain unaffected by the subsequent challenge with *Sp*, we observed a significant decrease of viral production in the context of *Sp* co-infection, notably at 8 hpi, with a more than three log10 difference of TCID50/mL in comparison with single RSV infection (Figure 2B).

To complete these observations, we also monitored the impact of RSV and *Sp* single and mixed infections on MDM viability (Figure 2C). Interestingly, we did not observe a marked difference between single and mixed infections, with a similar level of cell death around 20–30% (Figure 2C). Altogether, these results indicate that RSV particle release in supernatant is strongly reduced in the presence of *Sp*, suggesting a possible activation of macrophage that could contribute to mitigation of viral production.

RSV infection is associated with increased expression of various cytokines [29]. After characterizing the simple and mixed infections in macrophages, innate immune response to single or mixed RSV infection in MDM cells was examined by measuring changes in the expression of the CXCL10 gene. The mRNA levels of CXCL10 were measured in the cell culture of mock, single, or mixed infections at different time points after infection (Figure 2D). In our experimental conditions, *Sp* single infection did not induce the expression of IP-10, in contrast with our previously published study [6], most likely due to differences of protocols, which were adapted for RSV infection (e.g., lower cell confluency). In the context of single viral infection, we observed a dramatic increase in IP-10 expression at 14 hpi, with a fold change of more than 14,000 compared to the mock infection (Figure 2D). This high value could be explained by the relatively low level of IP-10 expressed in the mock infection (*C*_t_ value = 40) compared to the viral infection (*C*_t_ value = 27). Interestingly, this increase of CXCL10 expression was observed in the context of a very limited viral replication at the same time point (Figure 2B,D). A similar increase in CXCL10 expression was observed in the context of RSV/*Sp* mixed infection, but much earlier, at 8 hpi (Figure 2D). This observation suggests co-infection induces an innate immune response more rapidly. To further investigate the possible synergy that could occur in terms of activation of IP10 expression in response to mixed infections, we infected MDMs with two different MOIs of RSV (MOI 1 and 10) together with four different concentrations of *Sp* (0, 0.1, 1, and 10 CFU/cell) and monitored CXCL10 expression by RT-qPCR (Figure 2E). As expected, IP-10 expression was stimulated with an increasing quantity of viral inoculum. At an MOI of 1, CXCL10 expression was strongly induced in the presence of *Sp* (Figure 2E). Interestingly, at an MOI of 10, we observed a stronger increase of CXCL10 expression in the context of *Sp* co-infection (Figure 2E). Altogether, these results suggest that *Sp* could contribute to a faster and possibly higher level of CXCL10 expression induced by RSV infection in human MDMs.

## 3. Materials and Methods

### 3.1. Cell Lines and Culture Conditions

Hep-2 cells (ATCC CCL-23) were maintained in Eagle’s minimal essential medium (EMEM) with 2 mM l-glutamine and 10% FBS and maintained at 37 °C, 5% CO_2_. THP-1 MDMs were grown in RPMI 1640 medium l-glutamine containing 10% FBS and maintained at 37 °C, 5% CO_2_. THP-1 cells (ATCC TIB-202) were induced to differentiate into macrophages by incubation with 10 ng/mL phorbol myristate acetate (PMA; Invivogen, Toulouse, France) for 72 h.

### 3.2. Monocyte Isolation and Differentiation

Monocytes were isolated from human peripheral blood mononuclear cells (PBMC) and differentiated into macrophages as previously described [6].

### 3.3. Pathogen Preparation

RSV long strain (ATCC VR-26) was propagated in HEp2 cells. Encapsulated *Streptococcus pneumoniae* serotype 14 was obtained from the National Reference Center for *Streptococci* (Department of Medical Microbiology, Aachen, Germany). *Streptococcus pneumoniae* was cultured in the brain and heart-infused medium (BHI) according to the manufacturer’s instructions. Single colonies were expanded by resuspension in Todd-Hewitt and incubation at 37 °C for 3–4 h to the logarithmic phase. Bacteria were harvested by centrifugation at 1500× *g* for 15 min at 4 °C. Bacteria were then resuspended in cell culture medium at 1 CFU/cell.

### 3.4. Infection of Monocyte-Derived Macrophages (MDM) Cells

Cells were exposed to RSV Long at different multiplicity of infection (MOI) to investigate the kinetics of infection over time. Uninfected control replicates were exposed to cell culture medium. Fresh growth medium was applied, and cells were infected with bacterial strains at a different multiplicity of infection (MOI). *Pneumococci* were opsonized in anti-pneumococcal immune serum and incubated for 30 min at 37 °C prior to infections for all experiments. MDM cells showed significant loss of viability following overnight incubation with *S. pneumoniae*, so gentamicin (10 µg/mL) was added to all wells at the 2 h time point to prevent bacterial overgrowth and loss of cell viability.

### 3.5. Multiplex Real-Time RT-qPCR

Multiplex real-time RT-qPCR was performed to quantify RSV (F fusion gene) and *Sp* (autolysin A gene, LytA) in MDMs. The RT-qPCR reaction was performed using the AgPath-ID™ One-Step RT-PCR kit (Life Technologies; Carlsbad, CA, USA) according to the manufacturer’s instructions. Samples with a cycle threshold (*C*_t_) value of ≥40 were recorded as negative. A standard curve was prepared using serially diluted RNA extracts from a known quantity and used to calculate genomic copies/mL.

### 3.6. IP-10 mRNA Expression

For real-time quantitative PCR, total RNA was extracted using the RNAeasy Mini Kit (Qiagen, Valencia, CA, USA). Reverse-transcription was performed on 1 µg of total RNAs using the Superscript II enzyme (Invitrogen, Carlsbad, CA, USA) at 42 °C. Quantification of IP-10 mRNA levels was performed by real-time quantitative PCR using specific primers for IP-10; (F: 5′-GTGGCATTCAAGGAGTACCTC-3′, R: 5′-TGATGGCCTTCGATTCTGGATT-3′) and β-Actin (F: 5′-CTCTTCCAGCCTTCCTTCCT-3′, R: 5′-AGCACTGTGTTGGCGTACAG-3′) for normalization.

### 3.7. Apoptosis Assay

Cells were centrifuged and resuspended to be incubated with Annexin V-APC and propidium iodide (PI) (BD Biosciences, San Jose, CA, USA) for 15 min. Cells were analyzed by flow cytometry (Accuri C6; BD Biosciences) and the data visualized using FlowJo 7.6.5 software (FlowJo LLC, Ashland, OR, USA).

### 3.8. Immunofluorescence Microscopy

Infected cells were washed with PBS, fixed with 4% paraformaldehyde for 20 min at room temperature, permeabilized with PBS-0.1% Triton X-100, and incubated with PBS containing 1% BSA and 3% human serum to block non-specific binding. Reagents used for immunofluorescence in this study were goat anti-RSV (ab20745; Abcam, Cambridge, UK). Antibody incubations were performed for 2 h, followed by three washes with PBS. Bound RSV primary antibodies were detected using FITC conjugated donkey anti-goat antibody (ab6881; Abcam). Cells were imaged using a Leica DMI 3000B microscope equipped for fluorescence imaging. Images were rendered by ImageJ Software version 1.51 (NIH, USA).

### 3.9. Ethical Statement

Blood donation was obtained from healthy adult volunteers (Etablissement Français du sang, Lyon Gerland, France). Written informed consent from each subject was obtained (national procedure used for blood donations).

## 4. Conclusions

In conclusion, we showed that human MDMs constitute an interesting and biologically relevant experimental model for studying RSV/*Sp* co-infections. Our data support that early stages of RSV infection strongly trigger the innate immune response in human macrophages cells, in good agreement with previously published studies [29,42]. For example, Levitz and colleagues have recently described a strong induction of IL-6 and CCL5 in MDMs infected by different clinical isolates of RSV [42]. In a mice model, CXCL10 was demonstrated to have an important protective role to the host by reducing viral load and pathogenesis [43]. In the context of mixed infection, we have shown that expression of CXCL10 occurred earlier than in single RSV infection (16 h delay, Figure 2E), suggesting a faster and possibly stronger response of macrophages, also with good agreement with other in vitro and in vivo studies [44]. Interestingly, Vissers and colleagues have demonstrated that inactivated RSV induce CXCL10 expression [45]. Future investigations, with additional controls together with a higher number of data points, supported by statistical analyses (the lack of such analyses being a limitation of our study), will be necessary to complete our preliminary observations and to dress a more complete picture of RSV/*Sp* co-infections in MDMs.

We have shown that CXCL10 expression was strongly increased during infection, even if the level of viral replication appears very limited in MDMs. Based on this, we can speculate that strategies to control the immune response could constitute an interesting option in the treatment of patients with RSV infection. In this study, although RSV infection strongly mitigated by macrophages after 24 hpi, we have shown that bacterial co-infection contributes to alter the timing and extent of innate immune response, as illustrated by CXCL10 expression. We previously obtained quite similar results in a model of influenza/*Sp* co-infection infection in human MDMs, suggesting that common underlying mechanisms in different context of viral infection [6].

Future investigations may lead to the identification of key cell signaling pathways that mediate the host antiviral response to infection, which in turn could lead to the identification of novel drug targeting pro-inflammatory responses during infection by RSV. With increasing evidence of interactions between RSV and bacteria in the respiratory tract, it is important to better understand interconnections between pathogens and their combined effect on disease severity to develop future strategies for the prevention and treatment of RSV-induced severe acute LRIs.

## Abbreviations

RSV	Respiratory syncytial virus
*Sp*	*Streptococcus pneumoniae*
MDMs	Monocyte-derived macrophages
LRIs	Lower respiratory infections
IP-10	Interferon-γ-induced protein 10
